# Higher pathogen load in children from Mozambique vs. USA revealed by comparative fecal microbiome profiling

**DOI:** 10.1038/s43705-022-00154-z

**Published:** 2022-08-18

**Authors:** Minjae Kim, Luis M. Rodriguez-R, Janet K. Hatt, Osman Kayali, Rassul Nalá, Anne L. Dunlop, Patricia A. Brennan, Elizabeth Corwin, Alicia K. Smith, Joe Brown, Konstantinos T. Konstantinidis

**Affiliations:** 1 School of Civil and Environmental Engineering, Georgia Institute of Technology, Atlanta, GA 30332 USA; 2 Department of Microbiology and Digital Science Center (DiSC), University of Innsbruck, 6020 Innsbruck, Tyrol Austria; 3 School of Biological Sciences, Georgia Institute of Technology, Atlanta, GA 30332 USA; 4 Instituto Nacional de Saúde, Marracuene, Mozambique; 5 Department of Gynecology and Obstetrics, Emory University School of Medicine, Atlanta, GA 30332 USA; 6 Department of Psychology, Emory University, Atlanta, GA 30322 USA; 7 School of Nursing, Columbia University, New York, NY 10032 USA; 8 Department of Gynecology and Obstetrics, Department of Psychiatry and Behavioral Sciences, Emory University, Atlanta, GA 30322 USA; 9 Department of Environmental Sciences and Engineering, Gillings School of Global Public Health, University of North Carolina at Chapel Hill, North, Carolina, NC 27599 USA; 10 Present Address: Natural Resource Ecology Laboratory, Colorado State University, Fort Collins, CO 80523 USA

**Keywords:** Microbiome, Public health

## Abstract

The infant gut microbiome has lifelong implications on health and immunity but there is still limited understanding of the microbiome differences and similarities between children in low- and middle-income countries (LMICs) vs. high-income countries (HICs). Here, we describe and compare the microbiome profile of children aged under 48 months in two urban areas: Maputo, Mozambique and Atlanta, USA using shotgun metagenomics. The gut microbiome of American children showed distinct development, characterized by higher alpha diversity after infancy, compared to the same age group of African children, and the microbiomes clustered separately based on geographic location or age. The abundances of antibiotic resistance genes (ARGs) and virulence factors (VFs) were significantly higher in Maputo children, driven primarily by several primary and opportunistic pathogens. Most notably, about 50% of Maputo children under the age of two were positive for enterotoxigenic (ETEC) and typical enteropathogenic (EPEC) *Escherichia coli* diagnostic genes while none of the Atlanta age-matched children showed such a positive signal. In contrast, commensal species such as *Phocaeicola vulgatus* and *Bacteroides caccae* were more abundant in Atlanta, potentially reflecting diets rich in animal protein and susceptibility to inflammatory diseases. Overall, our results suggest that the different environments characterizing the two cities have significant, distinctive signatures on the microbiota of children and its development over time. Lack of safe water, sanitation, and hygiene (WASH) conditions and/or unsafe food sources may explain the higher enteric pathogen load among children in Maputo.

## Introduction

Over the past two decades, numerous studies investigated the role of the human microbiome, especially with respect to how dysbiosis of microbiota is related not only to infectious diseases but also to non-infectious diseases such as diabetes, asthma, obesity, and cardiovascular disease. To date, most microbiome research has focused on humans residing in high-income countries (HICs). There have been comparatively far fewer studies focused on low- and middle-income countries (LMICs), with most of the latter studies focused on East Asia [1]. Research on the microbiome of human populations living in other regions of the world such as South America, Africa, and South Asia (e.g., India) has been rarely conducted despite the unique cultural, lifestyle, and dietary diversity found in these regions [2–5] and the relatively high prevalence of gut infections that may have short- and/or long-term effects on health and development of children [1, 6, 7]. A few recent large-scale metagenomics surveys have revealed significant differences in taxa between human populations in HICs vs. LMICs [8, 9], but these studies were not based on age-matched cohorts, and thus the effects of factors such as age on the differences observed remain speculative. Although the effect of age on the development and inter-person variability of the gut microbiome has been well-recognized by now [5], a quantitative view of the diversity of the human microbiome around the world remains incomplete, especially for children in Africa [1].

Among the understudied regions, Africa has recently undergone rapid industrialization together with economic expansion resulting in profound changes in disease epidemiology, urban settlement, and population demographics [10]. In rapidly urbanizing, low-income settings of sub-Saharan Africa, food insecurity and poor hygiene, and lack of proper sanitation facilities increase the risk of infectious diseases, including diarrheal diseases caused by enteric pathogens [11]. Notably, several recent studies in this region have reported no significant associations between improved water, sanitation, and hygiene (WASH) conditions and enteric infections [12, 13]. On the other hand, the effects of WASH conditions on enteric infections can be complex and not easy to quantify due, for instance, to the lack of precision in WASH outcome measures, including self-reported (as opposed to objectively measured) diarrhea or asymptomatic carriage of enteric pathogens [14]. Further, comparison of data from different areas without easy ways to account for the differences such as rural vs. urban settings could be problematic [15].

Diarrheal diseases are the fifth leading cause of death among children under the age of 5 in LMICs, primarily due to unsafe water and sanitation [16], and can also affect the development of the gut microbiome. The gut microbiome is shaped in the first few years of life and is affected by several known factors such as the method of delivery, exposure to antibiotics, breastfeeding status, sanitation, and diet [17]. The interplay between these factors and diarrheal infections during development remains essentially poorly understood, especially in LMICs. A few recent studies investigated the effect of various factors on the gut microbiome in the early stage of life and compared between African and Western gut microbiomes. For example, Malawian infants showed higher abundances of *Bifidobacterium*, *Clostridium histolyticum*, and the *Bacteroides-Prevotella* group than did Finnish infants at 6 months of age [18]. Collectively, these previous studies have indicated that there might be significant differences in the gut microbiome of LMICs vs. HICs children that could be related to host phenotypes and development later in life [1]. However, the data and number of samples available for African populations are still limited for robust conclusions to emerge, particularly in terms of the temporal changes in the microbiome during the first 3–4 years of life (development), and the effect of geographic location which is often accompanied by different lifestyles, sanitation infrastructures, and diet. Furthermore, the majority of previous microbiome studies have focused on the comparison of pathogen burden between healthy and unhealthy children in LMICs [8, 19] and have not provided a comparative view, and possibly new insights, relative to age-matched children in HICs.

To provide new insights into these issues, we characterized the fecal microbiota of 153 children aged under 48 months living in urban settings of Maputo, Mozambique [UN human development index (HDI) of 0.456 classified as low human development [20]] as part of the Maputo Sanitation (MapSan) trial [21, 22] and compared these with the gut microbiota of 60 age-matched children living in Atlanta, USA [HDI of 0.9262 classified as very high human development], as part of the Emory University African American maternal stress and infant gut microbiome cohort study [23]. Our comparisons revealed significant differences in the intestinal microbial community structures between children from the two cities, some of which are most likely related to increased health risks for the Maputo children.

## Materials and methods

### Cohort description

The MapSan trial was a controlled before-and-after trial designed to evaluate the impact of an onsite sanitation intervention on child health after 12 and 24 months of follow-up [21]. Briefly, the intervention consisted of pour-flush toilets to septic tanks with soakaway pits to discharge the liquid portion of the waste. Control compounds did not receive the intervention and continued use of existing low-quality sanitation for the duration of the study. Participants included in this report (*n* = 177) are a subset of MapSan trial participants and we received written informed consent from a parent or guardian, and the head of the compound provided verbal assent for the compound to be included in the study [22].The difference in microbiome composition due to intervention were observed to be rather minor, and will be presented elsewhere as we focused here on the direct comparison between Maputo and Atlanta samples. The MapSan study protocol was approved by the Comité Nacional de Bioética para a Saúde (CNBS), Ministério da Saúde (333/CNBS/14), the Research Ethics Committee of the London School of Hygiene & Tropical Medicine (reference # 8345), and the Institutional Review Board of the Georgia Institute of Technology (protocol #H15160). Clinical trial registration ClinicalTrials.gov, number NCT02362932 [22].

For the Atlanta samples, we included a subset (*N* = 60) of the Emory University African American Maternal Stress and Infant Gut Microbiome Cohort Study [24]. In this study, pregnant women of African-American decent are enrolled during their first trimester of pregnancy and followed through delivery, completing an assembly of stress and behavioral measures at enrollment and again during the third trimester of pregnancy. At the third trimester data collection, the women are asked if they are interested in continuing as part of a postnatal mother-infant dyad cohort: those who agree are consented at that time and contact is continued through email and text messaging, until the infant is born. Upon birth of their infant, mothers again provide informed consent for inclusion of their infant in the postnatal study. The study was approved by the Emory University Internal Review Board (IRB), study ID is IRB00080193, and the appropriate review councils for each hospital where prenatal recruitment occurs [23].

### Stool collection and DNA sequencing

Stool samples from Atlanta children were obtained by the mother using Catch-All swabs. A small amount of stool (1–2 g) was collected and then placed into a pre-labeled hard plastic case for storage in the home freezer until collection by the study team within 72 h. Research staff then transferred the samples into labeled MoBio tubes and stored the tube in a −80 °C freezer. At the time of DNA extraction, samples were defrosted. The collection methods for Maputo stool samples were described previously [22]. The only difference in the stool sample collection methods and processing for DNA sequencing between Atlanta and Maputo was storage conditions until the sample was stored at −80 °C. Specifically, Atlanta samples were stored in a home freezer and transferred to −80 °C within 72 h while Maputo samples were kept in cold conditions and transferred to −80 °C within 6 h. This difference is unlikely to affect our results on diversity metrics and pathogen prevalence because no sample was stored in the freezer beyond three days prior to the storage at −80 °C [25]. Furthermore, the same DNA extraction methods (i.e., MoBio DNA isolation kit) and DNA sequencing were used for both Atlanta and Maputo samples. Specifically, for the Maputo samples, ~0.1 g of each fecal sample was used for DNA extraction and processed using Section 7.9 of the standard Manual of Procedures as suggested by the Human Microbiome Project (HMP- http://hmpdacc.org/resources/tools_protocols.php). No rectal swabs for Maputo were used in this study.

For the both Atlanta and Maputo samples, DNA was extracted from a similar volume of stool sample, which was stored in the MoBio tubes, using the MoBio isolation Kit according to the manufacturer’s protocol. DNA quantification was achieved using Qubit dsDNA HS Assay Kit (Thermo Fisher Scientific). DNA libraries were prepared using the Nextera XT DNA library prep kit (Illumina, San Diego, CA) and sequenced on an Illumina HiSEQ 2500 instrument (High Throughput Sequencing Core, Georgia Institute of Technology) for 300 cycles (2 × 150 bp paired end run) for Maputo samples and an Illumina NovaSeq 6000 instrument (High Throughput Sequencing Core, Georgia Institute of Technology) (2 × 150 bp paired end run) for Atlanta samples. Adapter trimming and demultiplexing of the sequenced samples were carried out by the instrument.

### Sequence quality checking, trimming and assembly

The sequenced shotgun metagenome reads were trimmed and quality checked using SolexaQA with cut-off of phred score 20 [26]. Trimmed reads were then filtered by BMTagger to identify and remove human reads [27]. Non-human reads, when longer than 50 bps after trimming, were used in the subsequent analyses. Assembly of the short reads for each metagenome (no co-assembly was attempted) was performed using IDBA-UD [28] and only resulting contigs longer than 1000 bp were used for genome binning. Resulting contigs from the assembly using MaxBin, and their completeness and contamination were assessed with CheckM [29, 30].

### Assembly and population genome binning

Metagenomic reads were quality-trimmed, assembled into contigs and contigs were binned into metagenome-assembled genomes (MAGs) as previously described [31] and further documented in the Supplementary Material. Quality of the MAGs was calculated as “Quality = Completeness – 5 × Contamination”, and MAGs with a quality score above 50 were used for further analysis (high quality MAGs). The Microbial Genome Atlas (MiGA) webserver was used to determine the most likely taxonomic classification and degree of novelty (e.g., whether the MAG represented a new species, genus, or family, etc.) of the high quality MAGs against the classified species in NCBI’s prokaryotic genome and TypeMat databases [32]. MAGs were named with unique identifiers (e.g., ANIsp_numbers) followed by the closest relative of the MAG and the lowest taxonomic rank the two share according to the MiGA results (i.e., p: for phylum, c: class, o- order, f: family, g: genus, and s: species). For instance, ANIsp_015_f:Coriobacteriales was used for a MAG that represented a novel genus of the *Coriobacteriales* family based on the lowest rank (family in this case) with significant assignment (*p* value < 0.1) shared with its best match against MiGA’s database. GTDBtk was also used to further confirm the assignments by MiGA as well as identify (taxonomically) unclassified closely related MAGs available in GTDB [33].

To de-replicate the collection of MAGs obtained from our metagenomes, we applied a genome-aggregate average nucleotide identity (or ANI) cutoff of 95% and selected one MAG with the highest quality score as the representative of each resulting 95% ANI-based genomospecies. The relative abundances of MAGs and individual genes in all metagenomes were calculated by competitive read mapping and normalized as genome equivalents (GEs), i.e., what fraction of the cells in the sample carries the gene of interest, by normalizing the relative abundance by the metagenomic dataset size and the community average genome size of the microbial community using MicrobeCensus [34]. Additionally, for a more conservative estimate, we calculated the 80% truncated coverage (TAD80) for MAG or gene abundance using the BedGraph.tad.rb script of the Enveomics collection [35], which removes outlier genomic regions in terms of coverage such as the rRNA and other multi-copy or recently horizontally transferred genes. Differently abundant MAGs/genes from different groups of samples were identified by the Kruskal–Wallis test followed by Dunn’s post-hoc test with p-value adjustment for each MAG/gene with all combinations of comparisons for the three different age groups (*P*_adj_ < 0.05) based on the Benjamini–Hochberg method as implemented in the FSA package [36] in R v4.0.2.

Supplementary Material includes further details on how sequencing coverage, α- and β-diversity indexes of each metagenomic dataset were calculated and compared as well as how the *E. coli* genome phylogeny was reconstructed.

## Results

### Description of the samples and metagenomes

**Fig. 1:**
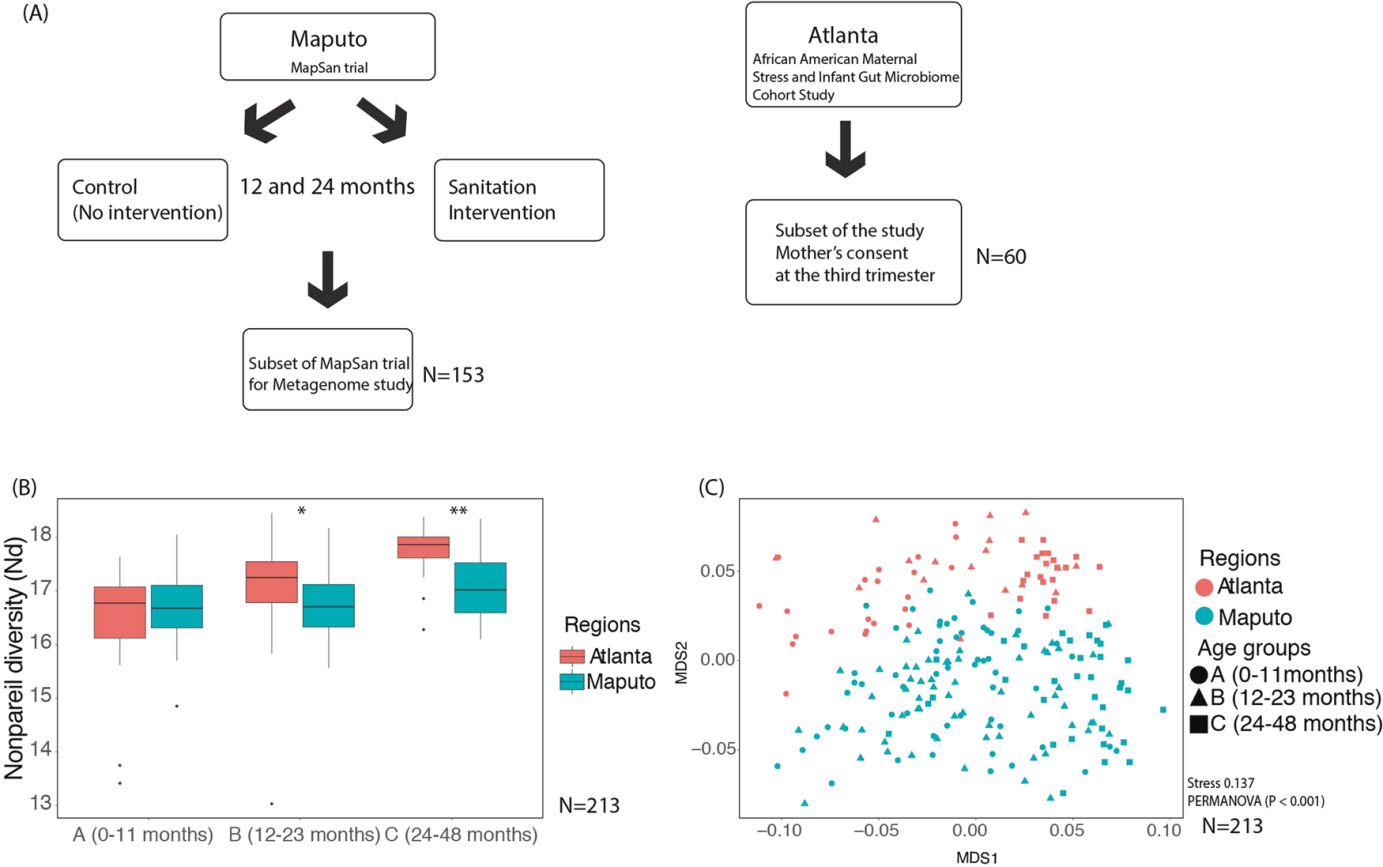
A flow-chart of the study design and microbial community diversity patterns between Atlanta and Maputo gut microbiomes. **A** A flow chart summarizing the samples analyzed from the MapSan trial and the African American Maternal Stress and Infant Gut Microbiome Cohort studies **B** Nonpareil diversity (*N*_*d*_) of the samples used in this study (*n* = 213) (*Significantly different at *P*_adj_ < 0.05, **P_adj_ < 0.01, Dunn’s post-hoc test). **C** A non-metric multidimensional scaling (NMDS) plot of microbial community similarity based on MASH distances of whole metagenomes, colored-code by city (Atlanta vs Maputo) and age (see key). Names starting with A denote the 0–11 month child group, B the 12–23 month group, and C the 24–48 month group (e.g., A_ATL indicates Atlanta children samples 0–11 months old in age).

A total of 177 Maputo stool samples were used for sequencing, assembly, and population genome binning. Out of these 177 samples, we used 153 for the comparisons due to the unknown age information for the remaining 24 samples. Fifty-eight of the latter samples were 0–11 month group, 61 were 12–23 month group, and 34 were 24–48 month group (Table 1 and Table S1). For Maputo stool samples, we attempted to select randomly but equal numbers of samples across two strata – age group and study arm (i.e., control vs. sanitation intervention)—at each study phase. Details on the study design for the MapSan trial can be found in the Materials and Methods section and in previous publications [21, 22] (Fig. 1A). Sixty Atlanta stool samples were sequenced to age-match the Maputo samples as follows: 22 were 0–11 month group, 17 were 12–23 month group, and 21 were 24–48 month group; the age of the participant for each sample can be found in Table S1 [23] (Fig. 1A).

**Table 1:** The number of samples and average/median age for each group.

# of samples	A: 0–11 months (Intervention^a^/Control)	B: 12–23 months (Intervention/Control)	C: 24–48 months (Intervention/Control)
Maputo, Mozambique	58 (32/26)	61 (27/34)	34 (15/19)
Atlanta, USA	22	17	21
Average/Median^b^	**A: 0–11 months (Average/Median)**	**B: 12–23 months (Average/Median)**	**C: 24–48 months (Average/Median)**
Maputo, Mozambique	9.33/9	16.75/16	29.32/29.5
Atlanta, USA	5.64/5.25	14.18/13	32.4/31

^a^Intervention indicates the samples from the sanitation intervention group and control indicates the samples from the control group in the Maputo Sanitation (MapSan) trial.

^b^Detailed information is in Table S1.

The estimated abundance-weighted average coverage calculated by Nonpareil, an algorithm that examines the extent of overlapping reads within a dataset to determine the coverage and diversity, ranged between 70 and 98% (Fig. S1), suggesting adequate coverage for genome binning and comparisons [37]. Nonpareil’s diversity metric (*N*_*d*_) represents the combined effect of richness and evenness (i.e., it represents total diversity) and coverage of 98% essentially means that there is 2% or less chance that the next sequenced read will represent a new sequence (i.e., be non-redundant with existing sequences). Assembly and genome binning efforts produced 1607 high-quality (completeness – 5*contamination ≥ 50) MAGs, 1333 from the Maputo datasets, and 274 from the Atlanta datasets. These MAGs represented 189 distinct genomospecies based on a 95% ANI threshold. Taxonomic classification through MiGA [32] suggested that 100/189 genomospecies showed less than 95% ANI to any reference genome of a previously described (known) species, indicating that these MAGs represent novel species or higher taxonomic ranks (Table S2). Specifically, 27 were predicted to be novel species of a previously described genus, 15 were novel species of a previously described family, and the remaining 58 represented novel families or higher taxonomic ranks of previously described phyla (Table S2).

Notably, out of these 100 novel genomospecies, 72 were recovered only from the Maputo samples, versus 28 from the Atlanta samples. However, it should be noted that taxonomic classification through GTDBtk [33] suggested that only 30 genomospecies were completely novel species that are not currently represented by available genomes, while the remaining matched previously recovered (largely unclassified at the species level) MAGs (Table S2).

### Lower diversity of Maputo children gut microbiome

Nonpareil sequence diversity (*N*_*d*_) values showed that the gut microbiome of Atlanta children was more diverse than that of the Maputo children in the 12–23 month group (*N*_*d*_ median values of 17.25 vs. 16.70, respectively; adjusted *P* value [*P*_adj_] of <0.05, Dunn’s post-hoc test; note that *N*_*d*_ is in log_e_ scale, so a difference of 1 unit corresponds to 2.7 fold difference) and 24–48 month group (*N*_*d*_ median values of 17.86 vs. 17.02, respectively; *P*_adj_ < 0.01, Dunn’s post-hoc test), while similar diversity was observed in the 0–11 month group (*N*_*d*_ median values of 16.77 vs. 16.68, respectively; *P*_adj_ ~ 0.85, Dunn’s post-hoc test) (Fig. 1B). Even though the Shannon diversity index based on 16S rRNA gene fragments recovered in the metagenomes was not significantly different between the two regions for the 0–11 and 12–23 month groups, Atlanta children in 24–48 month group showed higher microbiome diversity than Maputo children in the same age group as indicated by *N*_*d*_ (Fig. S2) and Shannon index (median values of 7.79 vs. 7.43, respectively; *P*_adj_ ~ 0.01, Benjamini and Hochberg correction applied). Note that Nonpareil diversity has been shown to be a more unbiased and sensitive method compared to traditional methods that use the taxa observed in a dataset as references such as Shannon diversity index because it is based on all reads of a dataset and is reference free [38]. Consistent separation of microbial communities among the three different age groups and two different regions was also obtained with Mash, a tool that uses kmer composition for β-diversity calculations (*R*^2^ value of 0.1274 for age and 0.1266 for region, and *P* value of <0.001, PERMANOVA) (Fig. 1C).

### Taxa responsible for lower diversity of Maputo microbiomes

To identify the prevalent taxa in our samples, the relative abundance (or just abundance hereon for simplicity) of the genomospecies was estimated using read recruitment plots and the TAD80 metric (Fig. S3). Notably, all 1607 MAGs together recruited an average of 54.5 ± 0.08% of the total reads in each metagenome (Fig. 3 and Fig. S4), revealing that the MAGs represent the majority of the microbial communities sampled and consistent with relatively high coverage values obtained by Nonpareil. In Atlanta samples, while several species such as *Escherichia coli* and *Bifidobacterium* spp. (*e.g., B. longum*, *B. bifidum*, and *B. breve*) were abundant in the 0–11 month group and became less abundant in 24–48 month group, other species such as *Bacteroides* spp. (e.g., *B. ovatus*, *Bacteriodes* sp. A1C1, and *B. caccae*), *Phocaeicola. vulgatus* (formerly known as *Bacteroides vulgatus*), *Faecalibacterium prausnitzii*, *Eubacterium rectale*, *Anaerostipes hadrus*, *Gemmiger formicilis,* and uncharacterized species of the *Ruminococcaceae* and *Lachnospiraceae* families, among others, became abundant or were exclusively found in the 24–48 month group (*P*_adj_ < 0.01, Dunn’s post-hoc test) (Fig. S3). These results are consistent with previous studies showing that *E. coli* and *Bifidobacterium* species are early colonizers of infant intestines and their abundance gradually drops in the adult gastrointestinal tract [17], while the relative abundance of *Bacteroidetes* and *Ruminococcaceae* increases as infants get older, e.g., around 3 years old [17].

**Fig. 2:**
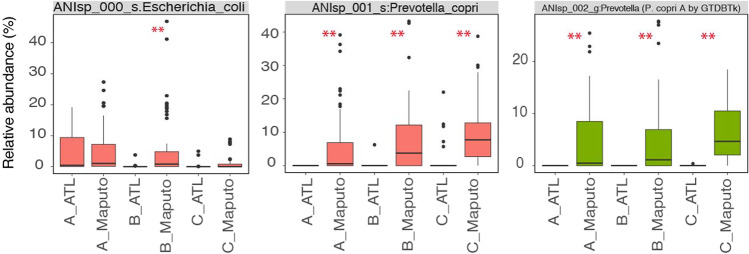
The relative abundance of *Escherichia coli* and two *Prevotella* species in Atlanta vs Maputo gut microbiomes. Labels on *x*-axis of each boxplot denote the age and region of the samples (sample names are as in Fig. 1). (Significantly different at **P_adj_ < 0.01, *P_adj_ < 0.05, Dunn’s post-hoc test). Results for all 52 genomospecies are available in Fig. S6 and Table S3.

Similarly, *E. coli* and *Bifidobacterium* spp. (*e.g., B. longum* and *B. breve*) were abundant in the 0–11 month group and became less abundant in the 24–48 month group in Maputo samples (Fig. S4) (*P*_adj_ < 0.01, Dunn’s post-hoc test). Interestingly, *Prevotella copri* (ANIsp_001_s:Prevotella_copri) became abundant, and even dominated the microbial community in the 24–48 month Maputo children by a median relative abundance of 7.8% of the total, while its relative abundance was only 0.6% in the 0–11 month children samples (*P*_adj_ < 0.01, Dunn’s post-hoc test) (Figs. 1 and S4). In addition to this highly abundant *P. copri* genomospecies, a closely related *Prevotella* genomospecies (ANIsp_002_g:Prevotella), showing 81% genome-aggregate average amino acid identity (AAI) to the dominant ANIsp_001, also accounted for a substantial part of the difference in beta-diversity, e.g., this genomospecies made up 4.6% of the total microbial community in the 24–48 month Maputo group (*P*_adj_ < 0.01, Dunn’s post-hoc test) (Fig. 2). Additional taxonomic classification via GTDBtk [33] suggested that these two (i.e., ANIsp_001 and ANIsp_002) are closely related to *Prevotella copri* (GCF_000157935.1; ANI of 95.33%) and *P. copri* A (GCF_002224675.1; ANI of 95.82%),which is different genomospecies from *P. copri* (e.g., <95% ANI), respectively. Therefore, *Prevotella* spp. accounted for a large part of the main difference observed in the diversity of the microbiome in Maputo vs. Atlanta samples of the two older age groups.

The differentially abundant taxa included 38 genomospecies that were not present in any of the Atlanta samples and 6 genomospecies that were not present in any of the Mozambique samples across all age groups (Fig. S5). Interestingly, these 38 genomospecies that were only observed in Maputo samples consisted largely of novel genomospecies as well as (previously) known commensal species including *Ligilactobacillus ruminis* and *Limosilactobacillus mucosae* (formerly known as *Lactobacillus ruminis* and *Lactobacillus mucosae*), and (opportunistic) human pathogens such as *Streptococcus pasteurianus* and *Brachyspira pilosicoli* that cause meningitis/bacteremia in newborns and human intestinal spirochetosis, respectively. The six species that were not present in any of the Maputo samples consisted mostly of novel species and a common human gut commensal species, i.e., *Acidaminococcus intestini*. The most notable difference however was the higher relative abundance of pathogenic and opportunistic pathogenic genomospecies in the Maputo samples that were virtually absent in Atlanta samples, which we explore further below. See also Supplementary Results for additional taxa detected as differentially abundant between the two sites and their potential functional roles.

### Prevalence of *E. coli* pathotypes in Maputo vs. Atlanta

**Fig. 3:**
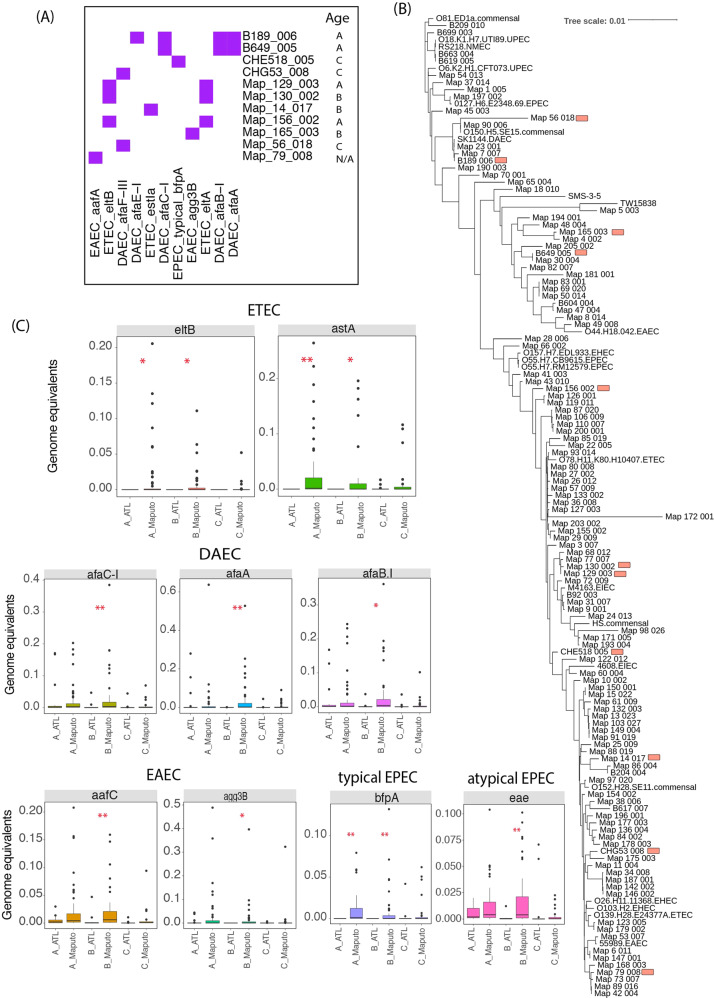
*E. coli* MAG pathotypes recovered in Maputo and Atlanta datasets. **A** A heatmap showing the presence (in purple) and absence (white) of diagnostic genes in the recovered *E. coli* MAGs. **B** Core genome phylogenetic tree of *E. coli* MAGs and selected reference genomes from the public databases. Red marked MAGs contain at least one of the *E. coli* diagnostic genes. Reference *E. coli* genomes include commensal strains (**HS commensal**, CP000802.1 strain HS; **O150H5SE15 commensal**, AP009378.1 strain SE15; **O152H28SE11 commensal**, AP009240.1 strains SE11; **O81ED1a commensal**, CU928162.2 strain ED1a), pathogenic strains (**0127H6E234869 EPEC**, FM180568.1 strain 0127:H6 E2348/69; **4608 EIEC**, gi|735003713|gb|JTCO01000001.1| strain 4608-58 4608-58_c1; **55989 EAEC**, gi|218350208|emb|CU928145.2| strain 55989; **M4163 EIEC**, gi|735003727|gb|JTCN01000001.1| strain M4163 M4163_c1; **O103H2 EHEC**, AP010958.1 strain O103:H2 str. 12009; **O139H28E24377A ETEC**, CP000800.1 strain E24377A; **O157H7EDL933 EHEC**, gi|749302083|ref|NZ_CP008957.1| strain O157:H7 str. EDL933; **O18K1H7UTI89 UPEC**, CP000243.1 strain UTI89; **O26H1111368 EHEC**, AP010953.1 strain O26:H11 str. 11368; **O44H18042 EAEC**, gi|284919779|emb|FN554766.1| strain 042; **O55H7CB9615 EPEC**, CP001846.1 strain O55:H7 CB9615; **O55H7RM12579 EPEC**, CP003109.1 strain O55:H7 RM12579; **O6K2H1CFT073 UPEC**, AE014075.1 strain CFT073; **O78H11K80H10407 ETEC**, FN649414.1 strain ETEC H10407; **RS218 NMEC**, CP007149.1 strain RS218; **SK1144 DAEC**, NZ_AP018784.1 strain SK1144), and environmental strains (**SMS35**, gi|170517292|gb|CP000970.1| strain SMS-3-5; **TW15838**, gi|329753645|gb|AEJX01000001.1|*E. sp*. TW15838). **C** *E. coli* diagnostic gene relative abundance (Significantly different at **P_adj_ < 0.01, *P_adj_ < 0.05, Dunn’s post-hoc test). Sample names are as in Fig. 1.

A total of 116 (104 from Maputo and 12 from Atlanta) of our recovered MAGs were identified as *E. coli* (>95% ANI to known *E. coli* genomes). The relative abundance of *E. coli* was higher in 12–23 month Maputo children compared to the Atlanta children (the median relative abundance of 0.83% vs. 0.00%, respectively; *P*_adj_ < 0.01, Dunn’s post-hoc test) (Fig. 2). At least four *E. coli* pathotypes, including enteroaggregative *E. coli* (EAEC), enterotoxigenic *E. coli* (ETEC), diffusely adherent *E. coli* (DAEC), and typical enteropathogenic *E. coli* (EPEC), were identified among these MAGs by searching their encoded genes against diagnostic pathotype-specific genes and their phylogenetic placement into a core gene alignment of known *E. coli* genomes that includes ETEC, EPEC, EAEC, uropathogenic *E. coli* (UPEC), DAEC, enterohaemorrhagic *E. coli* (EHEC), enteroinvasive *E. coli* (EIEC), neonatal meningitis-causing *E. coli* (NMEC), commensal, and environmental *E. coli* (Fig. 3). While not all these MAGs possessed the pathotype diagnostic genes, 11 did, and for the great majority of the remaining MAGs, the genes were detected in the metagenome (Supplementary Results) but apparently not binned into the MAG due to the genes being located in plasmids and other mobile elements [39]. Notably, we observed higher prevalence of ETEC diagnostic genes (i.e., *eltB*; enterotoxin subunit B and *astA*; heat-stable enterotoxin 1) in the 0–11 month (30 positive samples out of 58 samples for at least one ETEC diagnostic gene; or 52%) and 12–23 month (27 positive samples out of 61 samples; or 44%) groups in the Maputo samples relative to age-matched Atlanta samples (i.e., not present in any Atlanta sample). Not only prevalence, but their relative abundance was also higher in Maputo samples (*P*_adj_ < 0.05, Dunn’s post-hoc test; see Fig. 3C). Similarly, *bfpA* (bundlin pilin protein), which is a diagnostic gene typical of EPEC, was absent in the 0–11 and 12–23 month Atlanta groups, while it was prevalent in the corresponding Maputo groups (34/58 and 29/61 positives, respectively). Also *bfpA*’s relative abundance was higher in Maputo samples (*P*_adj_ < 0.01, Dunn’s post-hoc test) (Fig. 3C). These results suggested that the pathogenic *E. coli* load was substantially higher, especially in 0–23 month Maputo children compared to their age-matched Atlanta children.

### Higher abundance of antibiotic resistance genes (ARGs) in Maputo

**Fig. 4:**
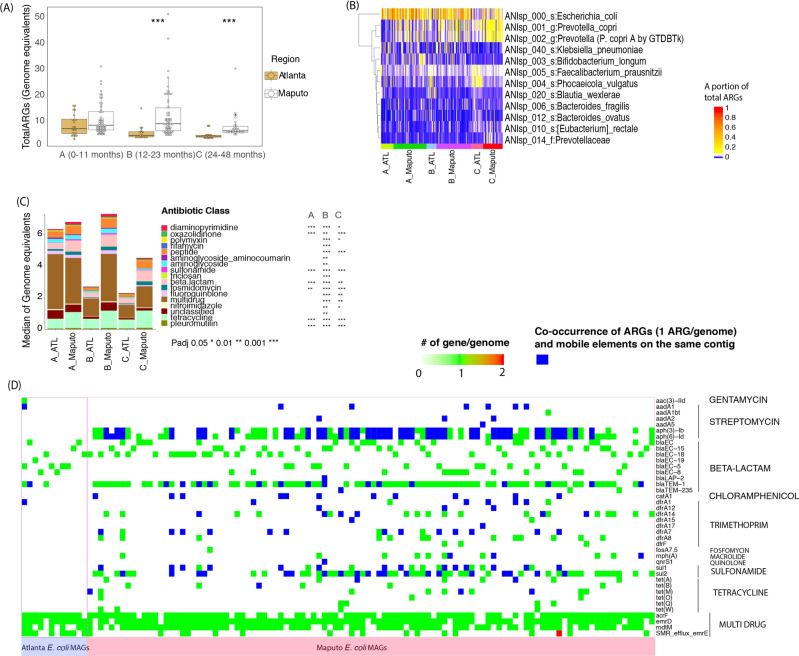
Antibiotic resistance gene (ARG) abundance and prevalence in children microbiomes. **A** total ARG abundance in Atlanta vs. Maputo datasets (figure key). **B** MAGs carrying most of the ARGs. Each raw represents a different genomospecies (taxonomic identity is provided on the right) and shows what fraction (figure key) of total ARGs in each sample (columns) is carried by the genomospecies. **C** Relative abundances (measured as Genome Equivalents or GEs; *y*-axis) of antibiotic resistance classes in each age group (*x*-axis) that showed significant differences in at least one age group comparison between the two locations. *P*_adj_ 0.05* 0.01** 0.001***, based on Dunn’s test. Sample names are as in Fig. 1. **D** Prevalence of ARGs in *E. coli* MAGs and their co-occurrence with mobile elements (integrons and transposons). For the complete description of each ARG protein shown, see Table S10.

Notably, both 12–23 and 24–48 month Maputo children showed significantly higher antibiotic resistance gene (ARG) abundances, measured as GEs, compared to their Atlanta counterparts by 2-fold and 1.6-fold, respectively (*P*_adj_ < 0.001, Dunn’s post-hoc test) (Fig. 4A). Taxonomic identification of the genomes carrying the ARG genes based on best match analysis of ARG-carrying reads against all MAGs recovered showed that *E. coli* carried the highest proportion of total ARGs in the 0–11 and 12–23 month groups (median of 16%, 29%, 7.5%, and 26% in 0–11 month of Atlanta, 0–11 month of Maputo, 12–23 month of Atlanta and 12–23 month of Maputo, respectively), followed by the *Prevotella* genomospecies mentioned above (Fig. 4B and Table S7). Therefore, it appears that most ARGs are carried by abundant microbiome members in Maputo. Several of the ARGs that were found to be more abundant in Maputo confer resistance specifically to the antibiotics recommended for use in children by the Mozambique Ministry of Health (e.g., ampicillin and gentamicin; see also Supplementary Results) (Figs. 4C and S7). Furthermore, we examined the potential for the mobilization of the clinically relevant ARGs, especially in *E. coli* MAGs, by assessing the co-occurrence of ARGs and mobile genes on the same contig. Based on a total of 116 *E. coli* MAGs (12 MAGs from Atlanta and 104 MAGs from Maputo samples), the number of ARGs per genome was significantly higher in the Maputo compared to the Atlanta MAGs (median value of 8 vs. 4 copies per genome, *p* value < 0.001, Kruskal-Wallis tests) (Fig. 4D). While the majority of the Atlanta *E. coli* MAGs only encoded beta-lactam and multi-drug resistance genes (e.g., only one Atlanta *E. coli* MAGs carried streptomycin, gentamycin, and trimethoprim resistance genes), many of the Maputo *E. coli* MAGs carried ARGs to several third-generation antibiotics such as streptomycin, chloramphenicol, trimethoprim, fosfomycin, macrolide, sulfonamide, and tetracycline resistance genes (Fig. 4D). Notably, many of these ARGs were co-occurring with mobile elements on the same contig. For example, 61 Maputo *E. coli* MAGs carried two streptomycin resistance genes (i.e., aminoglycoside O-phosphotransferase APH(3″)-Ib and aminoglycoside O-phosphotransferase APH(6)-Id) and 40 of these co-occurred with mobile elements on the same contig (Fig. 4D). Among the Maputo *E. coli* MAGs, 11 carried type A-1 chloramphenicol O-acetyltransferase, 14 MAGs carried sulfonamide-resistant dihydropteroate synthase *sul1*, and 20 MAGs carried *sul2* together with mobile elements, while none of the Atlanta *E. coli* MAGs encoded these genes. This result together with the virulent factors and *E. coli* pathotypes results described in the previous section further underscored the increased health risk for Maputo children.

Finally, among the additional species (e.g., other than *E. coli*), pathogens in the global priority list of antibiotic-resistant pathogens by the WHO (Fig. S8A and in ref. [40]) that included *Acinetobacter baumannii*, *Klebsiella pneumoniae*, *Enterobacter* spp., *Serratia* spp., *Proteus* spp., *Providencia* spp., *Morganella morganii*, *Enterococcus faecium, Staphylococcus aureus, Campylobacter* spp.*, Salmonella enterica, Streptococcus pneumoniae*, and *Haemophilus influenzae* were all detected in at least one metagenome but typically at relative low relative abundances. Most were detected in fewer than 5% of the total samples with the exception of *K. pneumoniae*, the *Enterobacter cloacae* complex, *M. morganii*, and *E. faecium*, which were detected in 119 (55.9%), 72 (33.8%), 16 (7.5%), and 27 (12.7%) metagenomes, respectively (Fig. S8B and Table S8; see also Supplementary Results). Even though we obtained positive signal for the presence of listed antibiotic-resistant pathogens, we were not able to determine if these genomes of actually carried specific antibiotic resistance determinants due to their low abundance which limited assembly and binning (unlike the *E. coli* populations mentioned above).

### Functional profile of the gut microbiome in Maputo vs. Atlanta

To compare the functional gene profiles of the gut microbiome between the two regions, we mapped metagenomic reads against the MetaCyc pathway database with Humann3. We detected a total of 481 pathways across all metagenomes. Out of 481 pathways, 150 pathways showed significant differences in abundance in at least one age group comparison between the two locations (*P*_adj_ < 0.01, Dunn’s post-hoc test) (Table S9). While the abundance of only 13 pathways was significantly different in 0–11 months, the abundance of 103 and 43 pathways showed significant differences in the 12–23 month and 24–48 month comparisons, respectively. This pattern was similar to that of ARGs gene abundances (Fig. 4A), and was overall in agreement with the *N*_*d*_ results (Fig. 1B) suggesting important differences in the development of infant gut microbiome between the two regions. Interestingly, out of 103 pathways that were differentially abundant in the 12–23 month comparisons, 97 pathways were enriched in Maputo samples, and the majority of these pathways were differentially abundant only in the 12–23 month comparisons and not the other age groups (Fig. S9 and Table S9). These enriched pathways included several distinct lipopolysaccharides synthesis (including polymyxin resistance), vitamin synthesis such as menaquinone (vitamin K2), tetrahydrofolate (vitamin B9) biosynthesis, etc.), and several amino acids and fatty acids biosynthesis pathways among others (Fig. S9 and Table S9). Additionally, adenosylcobalamin salvage from cobinamide I pathway and methanogenesis from acetate were enriched in all age groups of Atlanta samples compared to their counterparts. While the functional significance of these gene content differences remains to be elucidated more fully in the future, the findings are consistent with the taxon compositional differences revealed above, and suggest that the compositional differences are accompanied by significant functional gene content differentiation that is likely involved in the development of the microbiome and/or dietary differences. However, dietary records were not available for either cohort in our study to evaluate the effects of diet in more detail.

## Discussion

This study revealed several unique aspects of the gut microbiome of children aged under 48 months living in Maputo, Mozambique in comparisons with that of age-matched children living in Atlanta, USA. The β-diversity analysis suggested a clear separation of microbial communities among the three different age groups and two different regions and the reconstruction of MAGs showed about 70% of novel genomospecies were recovered from Maputo metagenomes. These results corroborate the findings of the recent studies that reported distinct and less-studied gut microbiota in African human populations compared to HICs populations [9, 18]. Furthermore, we observed the increase in α-diversity (i.e., *N*_*d*_) with age is much more pronounced in Atlanta vs. Maputo children microbiomes (*P*_adj_ < 0.0001, Dunn’s post-hoc test). Growth faltering in sub-Saharan Africa might be one of the major causes of poor development of gut microbiota in children aged 0–4 years old [41]. Interestingly, this finding contrasts to that of previous studies, which reported higher diversity in Malawian children older than three years of age relative to age-matched US children, and no significant difference in diversity between younger ones [5]. This difference could be due to the methods used (e.g., the previous study was based on error-prone 16S rRNA gene-amplicon data) or the different cohorts analyzed.

*Prevotella copri* was one of the major causes that differentiate the gut microbiome between the two regions studied by dominating the microbial community in the 24–48 month Maputo samples. Higher abundance of *Prevotella* is thought to be associated with the consumption of a fiber-rich diet (e.g., fruit and vegetables), while higher abundance of *Bacteroides* is usually linked to fat- and protein-rich diets [42, 43]; therefore, our findings likely reflect, at least in part, an effect of diet. Nonetheless our findings (Fig. 1B) contrast, at least partly, with previous findings reporting that higher fiber diet (in Africa) is related to higher alpha diversity, in addition to higher abundance of *Prevotella* spp. [2, 44], but the discrepancy may be due to the lack of comparisons among age-matched cohorts. Further, strain-level *P. copri* diversity (see also below) has been shown to be affected by diet (e.g., fiber-rich diets were linked to enhanced carbohydrate catabolism, while omnivore diet had a higher prevalence of genes -and strains- involved in branched-chain amino acid biosynthesis) [45]. Therefore, it appears that the functional consequences of the microdiversity of *Prevotella spp*. and related species could vary between healthy and non-healthy outcomes but overall remain poorly understood.

Our own results showed that ANIsp_001 (*Prevotella copri*) consists of MAGs recovered from both Maputo and Atlanta datasets, while ANIsp_002 (*Prevotella copri* A) only consists of MAGs recovered from Maputo datasets, which might suggest the presence of the geographically specific *Prevotella* sp. (Fig. 2 and Table S2). Due to the low number of *P. copri* MAGs recovered from the Atlanta samples (i.e., 5 MAGs) relative to Maputo (i.e., 88 MAGs), we were not able to perform a robust comparison of gene content differences between Atlanta and Maputo *Prevotella* population. For instance, it is likely that the *P. copri* populations represented by the Maputo MAGs may be present in Atlanta samples but below the limit of detection or sequencing depth required for robust assembly due -at least partly- to the higher diversity and/or lower number of Atlanta samples. Therefore, it would be interesting to study functional differentiation among the *P. copri* populations in the future, based on a larger Atlanta sample dataset. Despite this limitation, our preliminary results with the available MAGs suggested that there are tens of genes (29 for Atlanta *P. copri* MAGs and 12 for Maputo *P. copri* MAGs) that are specific to each group of *P. copri* MAG and those genes mostly encode for uncharacterized proteins, indicating that novel functions may be carried by these *Prevotella* populations.

In addition to the difference in commensal bacteria, we also observed the higher prevalence of pathogenic genomospecies in the Maputo samples. For example, we observed a higher prevalence of ETEC and typical EPEC in 0–23 month old Maputo children, while these *E. coli* pathotypes were absent in the age-matched Atlanta children. Our results offer quantitative insights into the pathogen load of children in a sub-Saharan African metropolitan area vs. a HIC city, and are consistent with previous findings from the Global Enteric Multicenter Study, which identified enterotoxigenic (ETEC) and typical enteropathogenic (EPEC) *E. coli* to be associated with increased risk of death in infants aged 0–11 months and show higher prevalence in sub-Saharan Africa and South Asia [6]. Functional gene analysis of the recovered *E. coli* MAGs revealed not only the higher frequency of ARGs in the Maputo MAGs but also the high potential for the horizontal transfer of such genes in Maputo (Fig. 4D).

It should be noted that 16S rRNA gene copy number of randomly selected subsamples for Maputo and Atlanta children (i.e., three for 0–11 months and 12–23 months, and four for 24–48 months for each region), as assessed by qPCR analysis, did not show significant difference between the selected Atlanta and Maputo samples (Kruskal-Wallis tests, *P* value > 0.1) (Fig. S10). This finding suggested that our results based on the relative abundance are robust and directly reflect absolute abundances. It should also be mentioned that there were 49 Maputo samples in our collection with positive detection of helminths, primarily *Ascaris* and *Trichuris* [22]. However, helminth infection did not seem to have a major impact on microbiome composition based on these samples (*R*^2^ value of 0.00911 with *P* value of >0.01, PERMANOVA) and thus, conclusions (assuming also no helminth infection for all Atlanta samples; helminth presence in Atlanta samples was not directly assessed by our study). Finally, the age distribution in the 0–11 month group was not even between Maputo and Atlanta cohorts. While 14 out of the total 22 Atlanta 0–11 month samples were in 0–6 month age range, contrasting with only 2 out of the 58 Maputo samples in the same age range (i.e., 8 Atlanta samples and 56 Maputo samples were in the 7–11 month age range). Therefore, we also compared the two regions using only subjects between 7 and 11 months old. We found that *Nd* values were still not significantly different between the two regions for 7–11 month (median values of 17.02 for Atlanta and 16.68 for Maputo; *P* value of 0.1384, Kruskal-Wallis tests). Thus, overall diversity does not appear to be substantially different between the two regions at the younger age, although more samples would be required for more robust conclusions in the future.

Collectively, our results revealed that pathogen load and asymptomatic infections in Maputo are highly prevalent and require action toward developing a healthier gut microbiome, which may support long-term health and well-being. Further, gut pathogens in this cohort are accompanied by a high abundance of ARGs, possibly related to the misuse or overuse of antibiotics in both humans and animals in this setting [46]. Lack of proper sanitation might be further promoting the spreading of ARGs and pathogens [47]. Future studies focusing on the relationship between environmental variables including WASH conditions, and exposure to antibiotics should be expected to provide further insights into effects of this relationship on the development of the gut microbiota of children.

## Limitations

Our study has limitations. Most notably, the sample size, while adequate for statistically significant comparisons, it is still probably limited in capturing the total diversity of the gut microbiome in both regions. It would be interesting to see if the patterns reported here apply to larger cohorts of children. Further, the most of the collected metadata other than age and region (e.g., breastfeeding, occurrence of helminth, protists, and enteropathogen based on sequence-independent means) were only available in one of cohorts (not the other) and/or where not reported systematically (e.g., antibiotic usage). This limited our assessment of the importance of these factors for the differences observed between the two regions.

## Supplementary information

Supplementary Methods

Table S10

## Data Availability

The biosamples used in this study are available in NCBI, under BioProject number PRJNA747761 (BioSample numbers SAMN20292687 to SAMN20292746 for Atlanta metagenomes and SAMN20292760 to SAMN20292936 for Maputo metagenomes). The MAG sequences recovered in this study are available under GenBank accession numbers JAIHOP000000000 – JAIJYJ000000000, as well as through http://enve-omics.ce.gatech.edu/data/atl_map_mags.
