# Supplementary material for: Higher pathogen load in children from Mozambique vs. USA revealed by comparative fecal microbiome profiling
Source: ISME Commun. 2022 Aug 18;2:74. doi: 10.1038/s43705-022-00154-z (PMC9723681; doi:10.1038/s43705-022-00154-z)
Supplement: Supplementary file 1 — Supplementary Methods [file 43705_2022_154_MOESM1_ESM.docx]

**SUPPLEMENTARY METHODS**

***Antibiotic Resistance Genes (ARGs), Virulence Factors (VFs), and metabolic pathways Identification***

To estimate the relative abundance of antibiotic resistance genes, DeepARG with short sequences model and default options (i.e., minimum probability of 0.8, identity of 50%, and alignment of 0.8) was used (Arango-Argoty et al 2018), which identifies ARG-encoding metagenomic reads after querying them against an internal ARG database. BLASTX search of metagenomic sequencing data against virulence factor database (VFDB) (Chen et al 2015) with the cutoff of bitscore 60 was used to calculate the relative abundance of virulence factors (VFs). Both ARG and VF relative abundances in metagenomes were converted to genome equivalents (GEs), i.e. what fraction of the cells in the sample carries the gene of interest, by normalizing for the metagenomic dataset size and the community average genome size of the microbial community using MicrobeCensus (Nayfach and Pollard 2015). Humann3 was used for pathway analysis (Beghini et al 2020). Pathway abundance was normalized to copies per million (cpm). Differently abundant ARG/VF genes and metabolic pathways between different groups of samples were identified by the Kruskal-Wallis test followed by Dunn’s post-hoc test with p-value adjustment based on the Benjamini-Hochberg method as implemented in the FSA package (Ogle 2016) in R v4.0.2. ARGs and mobile elements in *E. coli* MAGs were identified by BacAnt with default options (Hua et al 2021).

***Community Diversity Estimates***

Sequencing coverage and alpha diversity of each metagenomic dataset were calculated by Nonpareil version 3.20 with default parameters, an algorithm that examines the extent of overlapping reads within a dataset to determine the coverage and diversity (Rodriguez-r and Konstantinidis 2013). Pairwise distances of whole-community (*i.e.*, β-diversity) were calculated based on sample-to-sample kmer composition using MASH with the MinHash dimensionality reduction method (Ondov et al 2016). The MASH result was visualized using nonmetric multidimensional scaling (NMDS) plot with the vegan package in R v4.0.2 (Oksanen et al 2015). Permutational multivariate analysis of variance (PERMANOVA) was further used to reveal the effects of measured variables (i.e., age and location) on the MASH distances (Adonis function of the vegan package) (Oksanen et al 2015). Microbial community taxonomic composition was assessed based on clade-specific marker genes using MetaPhlAn3 (Beghini et al 2020), 16S rRNA gene-carrying reads, and metagenome-assembled genomes (MAGs). 16S rRNA gene (16S) fragments were recovered from shotgun metagenomes with Metaxa2 (v2.2) (Bengtsson‐Palme et al 2015). Sequences identified by Metaxa2 were subsequently searched using q2-vsearch in QIIME 2 for a closed reference picking approach with a 97% nucleotide identity cutoff for a match against SILVA-138 database (pre-clustered at 99% identity) (Bolyen et al 2019, Quast et al 2012, Sadaiappan et al 2021). The matching database reference sequences were used to calculate Shannon’s diversity index and compare the alpha diversity between the samples using QIIME 2 diversity analysis (Bolyen et al 2019).

***Phylogenetic Analysis***

Roary (Page et al 2015) was used to generate a core gene alignment of *Escherichia coli* MAGs together with selected available pathogenic, commensal, and environmental genomes of isolates that are available in the NCBI database (see detailed list of *E. coli* genomes in Figure 3). IQ-TREE version 2.0.3 (Minh et al 2020) was used to find the best model for building the whole core genome phylogeny, which was SYM+I+G4. RAxML-NG version 1.0.1 (Kozlov et al 2019) was used to construct a maximum-likelihood phylogeny based on this model and the core genome alignment.

**Quantification of 16S rRNA gene using qPCR**

DNA concentrations were measured using the Qubit high-sensitivity (HS) DNA kit (Thermo Fisher Scientific) and the Qubit 2.0 fluorometer. Template DNA samples were then diluted to 0.5-5 ng/μl before quantitative PCR (qPCR) amplification. The standard plasmid with full-length 16S rDNA gene was prepared using the TOPO-TA cloning kit (Invitrogen) as described previously (Suttner et al 2021). Six 10-fold serial dilutions (10^7^ to 10^2^ copies per reaction) were used to construct the standard curve of qPCR assays. All reactions were performed on the Applied Biosystems 7500Fast machine using the Custom TaqMan MGB probe (Applied Biosystems). The reaction mixture of the total volume of 20 μl was prepared to contain TaqMan Environmental MasterMix 2.0 (Applied Biosystems), 2 μl DNA template, 300 nM of each primer (Bac1055YF-ATGGYTGTCGTCAGCT, Bac1392R-ACGGGCGGTGTGTAC) (Ritalahti et al 2006) and TaqMan MGB probe with 5` reporter dye and 3` non-fluorescent quencher attached with MGB moiety (FAM-TGGACAGCCACCGACGAGAGCCTGG-NFQ-MGB). The Thermal cycles of qPCR were 2 min at 50°C followed by 10 min at 95°C and then 40 cycles of 15 seconds denaturation at 95°C followed by annealing/elongation for the 60s at 60°C as suggested by the manufacturer. The qPCR run included all samples and standard dilutions in triplicates. The quantification range was between 3,36.10^7^ – 3,36.10^2^ and all samples were within this range with standard curve R^2^:0.999. Sample copy numbers were also normalized with elution rates to give the results as copy numbers/μl of DNA sample.

**SUPPLEMENTARY RESULTS AND DISCUSSION**

***Gut microbiota differences between Atlanta and Maputo children***

To identify the key species that mostly contributed to the differentiation of the Atlanta vs. Maputo microbiomes, we conducted pairwise comparisons of the genomospecies relative abundance between the two cities, stratified by age (i.e., 0-11, 12-23, and 24-48 month groups). Fifty-two genomospecies, detected at both sites, including 24 known species and 28 novel species, showed significant differences in relative abundances (Figure 2, Figure S6, and Table S3) (P_adj_ < 0.01, Dunn’s post-hoc test). Notably, among the 52 genomospecies identified as differentially abundant in at least one of the comparisons, 19 species were assigned to the order *Bacteroidales* and 13 genomospecies to the order *Clostridiales* (Figure 2, Figure S6, and Table S3). The genomospecies that were more abundant in Maputo included, in addition to the two *Prevotella* species mentioned in the main text, several pathogenic or opportunistic pathogens that were virtually absent in the Atlanta samples (discussed further below). On the other hand, *Phocaeicola vulgatus* showed higher relative abundance in the microbiomes of the 24-48 month Atlanta children compared to the age-matched Maputo children (median relative abundance of 4.6% vs 0%, respectively; P_adj_ < 0.01, Dunn’s post-hoc test) (Figure S6).

Among the 13 *Clostridiales* species detected as differentially abundant between Maputo and Atlanta microbiomes, *Ruminococcus gnavus* showed higher relative abundance in the 12-23 month and 24-48 month Atlanta children compared to the Maputo children (median relative abundance of 0.02% and 0.004% in the Atlanta children vs. 0% in their Maputo counterparts, P_adj_ < 0.01, Dunn’s post-hoc test). Thus, *R. gnavus* showed similar relative abundance patterns with that of *Bacteroides* species (Figure S6), and this species has been implicated in Crohn’s disease (Henke et al 2019). Furthermore, *Lachnospiraceae*, especially *R. gnavus*, are early gut colonizers in infants (1 to 24 months of age), which is also consistent with our detection of this species in the 0-11 month Atlanta children but not in Maputo children (Figure S6) (Sagheddu et al 2016). *Anaerostipes hadrus*, which is an important butyrate-producing clostridial species in the human colon (Allen-Vercoe et al 2012), also showed higher relative abundance in the 12-23 and 24-48 month Atlanta children compared to Maputo children (the median relative abundance of 0.29% and 0.34% in 12-23 month and 24-48 month Atlanta children, respectively, vs. 0% in their Maputo counterparts, P_adj_ < 0.01, Dunn’s post-hoc test).

Among the remaining 20 species detected as differentially abundant between the two sites, *Escherichia coli* showed higher relative abundance in 12-23 month Maputo children compared to the Atlanta children (the median relative abundance of 0.83% vs. 0%, respectively; P_adj_ < 0.01, Dunn’s post-hoc test) (Figure 2). *E. coli* is an early colonizer of infant gut and its abundance supposed to be reduced as infant gets older. However, we observed *E. coli* remained abundant in the 24-48 month Maputo group and its higher relative abundance may be associated with the slower development of Maputo gut microbiomes relative to those of Atlanta children and/or infections (see also the main text about *E. coli* MAG analysis) (Nowrouzian et al 2003). *Haemophilus parainfluenzae*, which can cause several infectious diseases such as pneumonia, bloodstream infection, meningitis (https://www.cdc.gov/hi-disease/about/symptoms.html), also showed higher relative abundance in the 12-23 month Maputo children relative to its Atlanta counterpart (the median relative abundance of 0.28% in Maputo children samples, P_adj_ < 0.01, Dunn’s post-hoc test). However, *H. parainfluenzae* showed similar relative abundance (at 0.07% and 0.09%) in the 24-48 month Atlanta and Maputo children. Two distinct species of the order *Veillonellales* showed contrasting patterns in abundance, with ANIsp_013_f:Veillonellaceae showing higher relative abundance in the 0-11 month and the 24-48 month Maputo children (absent in all Atlanta samples, P_adj_ < 0.01, Dunn’s post-hoc test), while ANIsp_080_s:Dialister_invisus showing higher relative abundance in the 24-48 month Atlanta group (Figure S6). Taxonomic classification by GTDBtk suggested that ANIsp_013 and ANIsp_080 are closely related to *Dialister* sp000434475 and *Dialister invisus*, respectively. (Figure 2C). In addition to this, ANIsp_119 (*Flavonifractor* sp000508885 by GTDBTk) showed higher relative abundance in the 24-48 month Atlanta children than its counterpart Maputo group (median relative abundance of 0.05% vs. 0%, respectively; P_adj_ < 0.01, Dunn’s post-hoc test). Therefore, in addition to the primary pathogenic species mentioned in the main text (e.g., *E. coli*) several additional obligatory or opportunistic pathogens were detected in Maputo samples relative to their Atlanta counterparts (see also last section below), whereas the Atlanta samples included a higher relative abundance or prevalence of species correlated with western-diet-associated diseases.

***Prevalence of E. coli pathotype genes in Maputo vs. Atlanta***

To further corroborate the results of the phylogenetic assignments of *E. coli* MAGs mentioned in the main text, we assessed the presence/absence of diagnostic virulence factors (or VFs) of known *E. coli* pathotypes (Table S4). Interestingly, 11 MAGs (4 from Atlanta and 7 from Maputo) carried at least one of the diagnostic VFs, including aggregative adherence fimbria (EAEC), enterotoxins (ETEC), fimbrial adhesion operon (DAEC), bundle-forming pilus structural gene A (typical EPEC), and invasion protein (EAEC) (Figure 3A). Two of these *E. coli* MAGs (B189_006 and B649_005), reconstructed from the 0-11 month Atlanta group, encoded diagnostic genes of DAEC strains (i.e., afimbrial adhesion operon) and MAG B189_006 clustered with *E. coli* str. SK1144, a known DAEC strain, while another MAG (B649_005) clustered with several other *E. coli* MAGs but not close to any known pathogen, commensal, or environmental genome (Figure 3A and 3B). However, none of the other nine *E. coli* MAGs carrying the diagnostic genes were clustered with the *E. coli* reference pathogen genomes included here (Figure 3A and 3B), revealing that they represent novel genotypes (strains). In addition to these 11 *E. coli* MAGs carrying diagnostic genes, we also observed at least seven *E. coli* MAGs that were clustered together with known reference pathotypes of *E. coli* but did not encode diagnostic genes (Figure 3A and 3B). Since many of these diagnostic genes are known to be mobile (e.g., plasmid-borne) and the genome binning algorithms have known limitations in recovering such mobile elements, which typically have different copy number than the genome, it is likely that the diagnostic genes were not binned as part of the latter MAGs (as opposed to true gene absence). For instance, a recent study showed that *E. coli* MAGs with high (estimated) completeness (i.e., 95%) and only 1.5% of contamination still encoded only 77% of core genes and 50% of variable genes (Meziti et al 2021).

To identify cases where *E. coli* diagnostic genes were present in the sample but were not binned as part of the MAG or were wrongly binned (chimeric MAGs), we also compared the presence/absence and relative abundance of these genes based on the short metagenomic reads. Mapping short metagenomic reads against a virulence factor database (VFDB) revealed that virulence factor (VF) relative abundances were higher in both 12-23 and 24-48 month Maputo children by 2.5 fold and 1.72 fold, respectively compared to the Atlanta children (P_adj_ < 0.01, Dunn’s post-hoc test) (Table S6). Alignments of VF matched reads against MAGs identified that *E. coli* carried the highest proportion of the total vVFs detected in both the 0-11 and 12-23 month age groups (median of 19%, 26%, 7.3%, and 24% in 0-11 month Atlanta, 0-11 month Maputo, 12-23 month Atlanta, and 12-23 month Maputo, respectively), while *P. vulgatus* (median of 6.1%), and ANIsp_001 and ANIsp_002 (*Prevotella sp.* by GTDBtk) (median of 9.9% and 8.2%, respectively) carried the highest proportion of total VFs in the 24-48 month Atlanta and Maputo groups (Table S5). Additionally, two diagnostic genes, i.e., aggregative adherence fimbria II usher protein (*aafC*) and invasion protein (*agg3B*), for EAEC showed higher relative abundance in the 12-23 month Maputo group compared to its Atlanta counterpart (P_adj_ < 0.05, Dunn’s post-hoc test) (Figure 3C). Therefore, for the majority of MAGs identified as probable pathogens by phylogenetic placement (but did not carry the pathotype diagnostic genes), we were able to detect the diagnostic genes in the corresponding metagenomes, indicating that the genes were simply missed by the binning step. These results also agreed with a recent study reporting that enteroaggregative *E. coli* (EAEC) may be the leading cause of foodborne and water-borne enteric infections in South Africa (Aijuka et al 2018).

***Antibiotic resistance genes (ARGs) in Maputo***

A total of 31 different ARG classes were detected in the metagenomes, with multidrug, tetracycline, and MLS (macrolide, lincosamide, and streptogramin) showing the highest relative abundance in all metagenomes (median GE values: 2.1, 0.97, and 0.61, respectively) (Table S6). Further comparisons revealed that 17 distinct ARG protein families were differentially abundant between Maputo and Atlanta in at least one age group (Figure 4C) (P_adj_ < 0.01, Dunn’s post-hoc test). Specifically, beta lactamases showed a higher relative abundance in Maputo in all age groups (P_adj_ < 0.01, Dunn’s post-hoc test), and aminoglycoside showed a higher relative abundance in 12-23 month Maputo children relative to the same age group of Atlanta children (Figure 4C and S7) (P_adj_ < 0.01, Dunn’s post-hoc test). These results were likely driven, to a certain extent, by the recommendation of the Mozambique Ministry of Health to use ampicillin and gentamicin (i.e., aminoglycoside class) for treating children under the age of two, and chloramphenicol for older children (Center for Disease Dynamics 2015). Even though there was no significant difference in the relative abundance of reads mapping to phenicol resistance genes between the two locations (Table S6) (P_adj_ > 0.01, Dunn’s post-hoc test), the current recommendation for using ampicillin and gentamicin for children under 2 years old might be reevaluated due to the higher prevalence of the corresponding resistance genes in the gut of Maputo children. It would also be important to further validate the genome-based ARG results with functional (phenotypic) experiments prior to making changes in the use of antibiotics.

Additionally, taxonomic identification of the genomes carrying the ARG genes based on best match analysis of ARG-carrying reads against all MAGs recovered showed that *E. coli* carried the highest proportion of total ARGs in the 0-11 and 12-23 month groups (median of 16%, 29%, 7.5%, and 26% in 0-11 month Atlanta, 0-11 month Maputo, 12-23 month Atlanta and 12-23 month Maputo children, respectively) (Figure 4B and Table S7). *P. vulgatus* (median of 7.2%), ANIsp_001 (*P. copri*, median of 13%), and ANIsp_002 (*P. copri* A by GTDBTk, median of 11%) carried the highest proportion of total ARGs in the 24-48 month Atlanta and Maputo children (Figure 4B). This result was consistent with the high prevalence of *E. coli* in both Maputo and Atlanta 0-11 month children, and in the 12-23 month Maputo children compared to its Atlanta counterpart, and also consistent with decreased *E. coli* relative abundance in the 24-48 month groups of both locations (Figure 2 and S6). Therefore, the high prevalence of ARG-carrying *E. coli* underlaid the major difference in total ARG abundance between the two locations and the 12-23 month group (Figure 2 and S6). The higher relative abundance of *P. vulgatus* in the 24-48 month Atlanta children and the higher relative abundance of *P. copri* in the 24-48 month Maputo children compared to their age-matched counterparts supported the ARG results reported above (Figure 4B and Figure S6). Notably, *P. copri* and *P. copri* A (ANIsp_001 and ANIsp_002, respectively) were responsible for significant proportions of total ARGs in all age groups except the 0-11 month Atlanta group (median of less than 1% of total ARGs in the 0-11 month Atlanta and median of 1.8% to 12.72% of total ARG-carrying reads in the other two groups) (Figure 4B and 2). *Faecalibacterium prausnitzii* also carried a significant portion of total ARGs in all age groups (i.e., second highest in all age groups of the Atlanta samples and 4^th^ highest in all age groups of the Maputo samples, following the *E. coli* and the two *Prevotella* populations), ranging from a median of 2.4% to 6.4% (Figure 4B). This result implied the potentially important role of *F. prausnitzii* as an ARG reservoir in both African and American children.

***Surveillance of antibiotic resistant bacteria (WHO)***

Even though we reconstructed MAGs that can represent well the gut microbial community (e.g., ~54% reads recruited, on average), there were low-abundance species whose genomes were not recovered by our population genome binning efforts. To provide windows into this rare diversity, MetaPhlAn3 species profile, which uses clade-specific marker genes that can be covered by only a few reads for positive detection compared to the coverage required for genome binning (6-7X or more) (Meziti et al 2019), was also employed.

Among the pathogens mentioned in the global priority list of antibiotic-resistant pathogens by the WHO (Figure S8A and in (WHO 2017)), *Acinetobacter baumannii*, *Klebsiella pneumoniae*, *Enterobacter* spp., *Serratia* spp., *Proteus* spp., *Providencia* spp., *Morganella morganii*, *Enterococcus faecium, Staphylococcus aureus, Campylobacter, Salmonella enterica, Streptococcus pneumoniae,* and *Haemophilus influenzae* were all detected in at least one metagenome by MetaPhlAn3 (Table S8). While most of these pathogens were (individually) detected in fewer than 9 metagenomes out of a total 213 metagenomes used in this study (i.e., less than 5% of total), *K. pneumoniae*, the *Enterobacter cloacae* complex, *M. morganii*, and *E. faecium* were detected in 119, 72, 16, and 27 metagenomes, respectively (Figure S8B; for *E. coli*, see previous section). *K. pneumoniae* was the most prevalent antibiotic-resistant pathogen in both Maputo and Atlanta datasets, and its mean relative abundance did not differ between the two locations although its potential virulence content based on VFs and ARGs recovered in the corresponding MAGs was likely higher in Maputo (see previous section). The *Enterobacter cloacae* complex, including *E. asburiae*, *E. cloacae*, *E. hormaechei*, *E. kobei*, *E. ludwigii*, and *E. nimipressuralis,* is increasingly identified as nosocomial pathogens, causing 7% of nosocomial infections in intensive care units in the USA alone (Hoffmann et al 2005, Jones 2003, Sanders and Sanders 1997, Streit et al 2004). Interestingly, the marker genes for the *E. cloacae* complex were detected in 45%, 51%, and 29% of the samples from the 0-11 month, 12-23 month, and 24-48 month Maputo groups, respectively, while the same values for Atlanta samples were 23% for the 0-11 month group and under the detection limit for the two other Atlanta age groups (Figure S8B). The same pattern was observed for *M. morganii* that has been reported to be associated with several neonatal diseases such as neonatal meningitis, sulfhemoglobinemia, and hemolytic anemia (Figure S8B) (Milligan and Barenkamp 2013, Murphy et al 2015). *Enterococci*, especially *E. faecalis* and *E. faecium*, have become the most commonly isolated Gram-positive nosocomial pathogens around the world (Guzman Prieto et al 2016, Leavis et al 2006, Murray 1990). Especially, *E. faecium* infections have been dramatically increased in recent years and currently account for around 40% of all enterococcal infections (Gao et al 2018, Low et al 2001, Top et al 2008). The marker genes of *E. faecium* were detected in 14% and 24% of the samples of the 0-11 month Atlanta and Maputo groups, respectively, and remained high in the Maputo 12-23 month group, while the marker genes were not detectable in its Atlanta counterpart. There was no detectable *E. faecium* signal in both the Atlanta and Maputo 24-48 month groups. Overall, in addition to the abundant pathogens mentioned in the main text such as *E. coli*, several additional obligatory or opportunist pathogens were detected in the Maputo (but not the Atlanta) metagenomes at lower abundances.

**SUPPLEMENTARY FIGURES**

**Figure S1. Nonpareil curves.** Red curves represent the Maputo samples (n=177) and blue curves the Atlanta samples (n=60). The two circles represent the estimated average coverage of the microbial community sampled by the sequencing depth/effort applied. The projected line to the right of the circle represents the expected coverage for higher sequencing efforts. Dashed lines represent the sequencing needed for 95% and 99% coverage levels. Curves positioned more on the right represent more sequence-diverse metagenomes compared to curves positioned on the left.


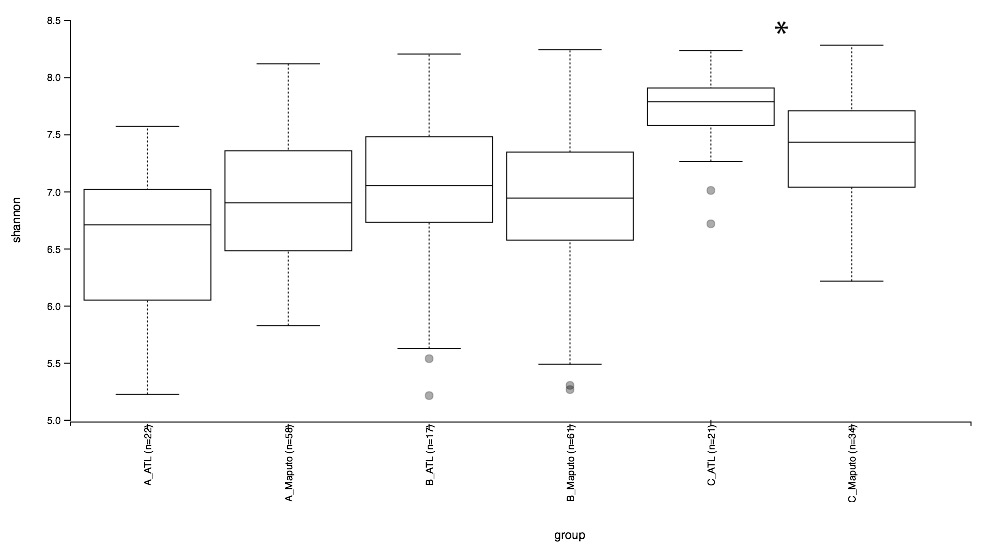


**Figure S2. Shannon diversity index based on the 16S rRNA gene fragments recovered from the Atlanta and Maputo metagenomes.** (Significantly different at *P_adj_ < 0.05, **P_adj_ < 0.01, **Dunn’s post-hoc test)**

**Figure S3. Relative abundance patterns of genomospecies (MAGs) in Atlanta children gut microbiomes determined in this study**. The graph represents a heatmap of the relative abundances (columns; see key) of each genomospecies (rows); empty/white cells denote that the MAG was not detected. The relative abundance was estimated based on the normalized TAD80 metric as described in the Materials and Methods section.

**Figure S4. Relative abundance patterns of genomospecies (MAGs) in Maputo children gut microbiomes determined in this study**. The graph represents a heatmap of the relative abundances (columns; see key) of each genomospecies (rows); empty/white cells denote that the MAG was not detected. The relative abundance was estimated based on the normalized TAD80 metric as described in the Materials and Methods section.


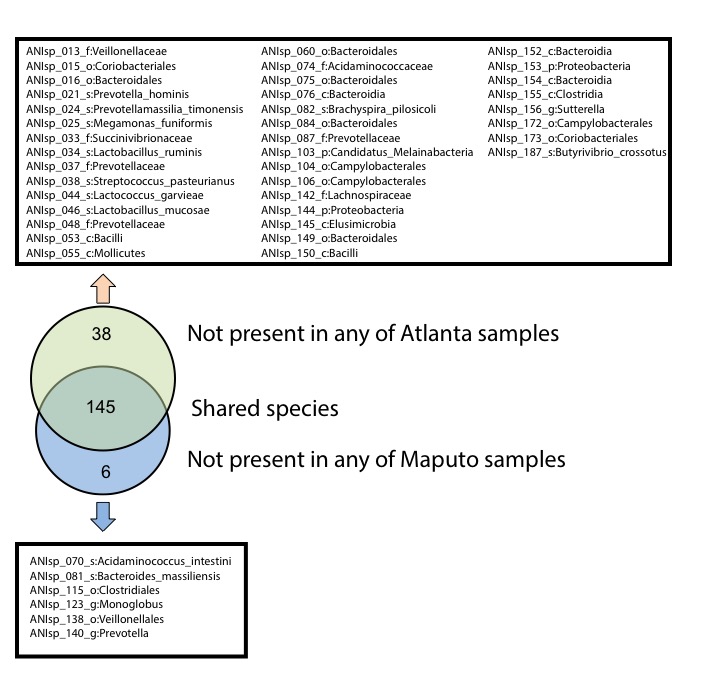


**Figure S5. A diagram representing presence and absence of MAGs in Atlanta vs. Maputo metagenomes.** MAGs were named with unique identifiers (e.g., ANIsp_numbers) followed by the taxonomic name of the lowest rank that the MAG shared with its closest relative in the MiGA database (i.e., p: phylum, c: class, o: order, f: famly, g: genus, and s: species). For instance, we used ANIsp_081_f:*Bacteroidaceae* for a MAG that had a novel species of *Bacteroidaceae* family as the closest relative and was classified at the lowest level with MiGA (p value < 0.1).

**Figure S6. Genomospecies that showed differential relative abundance between Atlanta and Maputo metagenomes in at least one of the age groups assessed.** (A) Genomospecies assigned to the order *Bacteroidales*. (B) Genomospecies assigned to the order *Clostridiales*. (C) Other genomospecies. Taxonomy classification information and detailed statistics for each MAG (genomospecies) is available in Table S8. (Significantly different at ** P_adj_ < 0.01, * P_adj_ < 0.05, Dunn’s post-hoc test)

**Figure S7. Differently abundant ARG classes detected in the Atlanta and Maputo metagenomes.** Reads identified by DeepARG were used to calculated genome equivalents as described in the Materials and Methods section. Statistically significant at P_adj_ 0.05 * 0.01 ** 0.001 ***, Dunn’s test.

**Figure S8. Antibiotic-resistant pathogens detected from the WHO list.** (A) The antibiotic-resistant pathogens in the global priority list of the WHO (adapted from (WHO 2017)). (B) Percent of antibiotic-resistant pathogens positive samples in each children group. Names starting with A denote the 0-11 month child group, B the 12-23 month group, and C the 24-48 month group (e.g., A_ATL indicates Atlanta children samples 0-11 months old in age).

**Figure S9. Functional pathways that differentiate Maputo and Atlanta cohorts.** Values indicate Z-score from Dunn’s test (* P_adj_ < 0.01). Blue and red colors indicate pathways enriched in Atlanta and Maputo samples, respectively. Group A denote the 0-11 month child group, Group B the 12-23 month group, and Group C the 24-48 month group. A full list of the functional pathways that are differentially abundant in at least one age group comparison is available in Table S9.

**Figure S10. qPCR of 16S rRNA gene.** The figure shows the microbial load (copy number of the 16S rRNA gene per μl of sample) as assessed by qPCR analysis of ten randomly selected samples from Maputo (Map_1, Map_104, Map_110, Map_121, Map_124, Map_153, Map_201, Map_51, Map_55, and Map_98) and Atlanta (B138, B266, B632, B655, B88, CHE518, CHE535, and CHG130). The metadata for each sample can be found in Supplementary table 1. qPCR analysis was run on an Applied Biosystems 7500Fast machine using the Custom TaqMan MGB probe (Applied Biosystems), essentially as described in (Ritalahti et al 2006) and detailed in the Supplementary Methods section.

**REFERENCES**

Aijuka M, Santiago AE, Girón JA, Nataro JP, Buys EM (2018). Enteroaggregative Escherichia coli is the predominant diarrheagenic E. coli pathotype among irrigation water and food sources in South Africa. *International Journal of Food Microbiology* **278:** 44-51.

Allen-Vercoe E, Daigneault M, White A, Panaccione R, Duncan SH, Flint HJ *et al* (2012). Anaerostipes hadrus comb. nov., a dominant species within the human colonic microbiota; reclassification of Eubacterium hadrum Moore et al. 1976. *Anaerobe* **18:** 523-529.

Arango-Argoty G, Garner E, Pruden A, Heath LS, Vikesland P, Zhang L (2018). DeepARG: a deep learning approach for predicting antibiotic resistance genes from metagenomic data. *Microbiome* **6:** 23.

Beghini F, McIver LJ, Blanco-Míguez A, Dubois L, Asnicar F, Maharjan S *et al* (2020). Integrating taxonomic, functional, and strain-level profiling of diverse microbial communities with bioBakery 3. *bioRxiv*.

Bengtsson‐Palme J, Hartmann M, Eriksson KM, Pal C, Thorell K, Larsson DGJ *et al* (2015). METAXA2: improved identification and taxonomic classification of small and large subunit rRNA in metagenomic data. *Molecular ecology resources* **15:** 1403-1414.

Bolyen E, Rideout JR, Dillon MR, Bokulich NA, Abnet CC, Al-Ghalith GA *et al* (2019). Reproducible, interactive, scalable and extensible microbiome data science using QIIME 2. *Nature biotechnology* **37:** 852-857.

Center for Disease Dynamics EP (2015). Global Antibiotic Resistance Partnership-Mozambique Working Group. 2015. Situation Analysis and Recommendations: Antibiotic Use and Resistance in Mozambique.

Chen L, Zheng D, Liu B, Yang J, Jin Q (2015). VFDB 2016: hierarchical and refined dataset for big data analysis—10 years on. *Nucleic acids research* **44:** D694-D697.

Gao W, Howden BP, Stinear TP (2018). Evolution of virulence in Enterococcus faecium, a hospital-adapted opportunistic pathogen. *Current opinion in microbiology* **41:** 76-82.

Guzman Prieto AM, van Schaik W, Rogers MR, Coque TM, Baquero F, Corander J *et al* (2016). Global emergence and dissemination of enterococci as nosocomial pathogens: attack of the clones? *Frontiers in microbiology* **7:** 788.

Henke MT, Kenny DJ, Cassilly CD, Vlamakis H, Xavier RJ, Clardy J (2019). Ruminococcus gnavus, a member of the human gut microbiome associated with Crohn’s disease, produces an inflammatory polysaccharide. *Proceedings of the National Academy of Sciences* **116:** 12672-12677.

Hoffmann H, Stindl S, Ludwig W, Stumpf A, Mehlen A, Heesemann J *et al* (2005). Reassignment of Enterobacter dissolvens to Enterobacter cloacae as E. cloacae subspecies dissolvens comb. nov. and emended description of Enterobacter asburiae and Enterobacter kobei. *Systematic and applied microbiology* **28:** 196-205.

Hua X, Liang Q, Deng M, He J, Wang M, Hong W *et al* (2021). BacAnt: a combination annotation server for bacterial DNA sequences to identify antibiotic resistance genes, integrons, and transposable elements. *Frontiers in Microbiology* **12**.

: Global epidemiology of antimicrobial resistance among community-acquired and nosocomial pathogens: a five-year summary from the SENTRY Antimicrobial Surveillance Program (1997-2001). *Seminars in respiratory and critical care medicine*.

Kozlov AM, Darriba D, Flouri T, Morel B, Stamatakis A (2019). RAxML-NG: a fast, scalable and user-friendly tool for maximum likelihood phylogenetic inference. *Bioinformatics* **35:** 4453-4455.

Leavis HL, Bonten MJ, Willems RJ (2006). Identification of high-risk enterococcal clonal complexes: global dispersion and antibiotic resistance. *Current opinion in microbiology* **9:** 454-460.

Low DE, Keller N, Barth A, Jones RN (2001). Clinical prevalence, antimicrobial susceptibility, and geographic resistance patterns of enterococci: results from the SENTRY Antimicrobial Surveillance Program, 1997–1999. *Clinical Infectious Diseases* **32:** S133-S145.

Meziti A, Tsementzi D, Rodriguez-R LM, Hatt JK, Karayanni H, Kormas KA *et al* (2019). Quantifying the changes in genetic diversity within sequence-discrete bacterial populations across a spatial and temporal riverine gradient. *The ISME journal* **13:** 767-779.

Meziti A, Rodriguez-R LM, Hatt JK, Peña-Gonzalez A, Levy K, Konstantinidis KT (2021). How reliably do metagenome-assembled genomes (MAGs) represent natural populations? Insights from comparing MAGs against isolate genomes derived from the same fecal sample. *Applied and Environmental Microbiology*.

Milligan KL, Barenkamp SJ (2013). Neonatal meningitis due to Morganella morganii. *Clinical pediatrics* **52:** 462-464.

Minh BQ, Schmidt HA, Chernomor O, Schrempf D, Woodhams MD, Von Haeseler A *et al* (2020). IQ-TREE 2: New models and efficient methods for phylogenetic inference in the genomic era. *Molecular biology and evolution* **37:** 1530-1534.

Murphy K, Ryan C, Dempsey EM, O’Toole PW, Ross RP, Stanton C *et al* (2015). Neonatal sulfhemoglobinemia and hemolytic anemia associated with intestinal Morganella morganii. *Pediatrics* **136:** e1641-e1645.

Murray BE (1990). The life and times of the Enterococcus. *Clinical microbiology reviews* **3:** 46-65.

Nayfach S, Pollard KS (2015). Average genome size estimation improves comparative metagenomics and sheds light on the functional ecology of the human microbiome. *Genome biology* **16:** 51.

Nowrouzian F, Hesselmar B, Saalman R, Strannegård I-L, Åberg N, Wold AE *et al* (2003). Escherichia coli in infants' intestinal microflora: colonization rate, strain turnover, and virulence gene carriage. *Pediatric research* **54:** 8-14.

Ogle DH (2016). *Introductory fisheries analyses with R*. Chapman and Hall/CRC.

Oksanen J, Blanchet FG, Kindt R, Legendre P, Minchin PR, O’hara R *et al* (2015). Package ‘vegan’. *Community ecology package, version* **2**.

Ondov BD, Treangen TJ, Melsted P, Mallonee AB, Bergman NH, Koren S *et al* (2016). Mash: fast genome and metagenome distance estimation using MinHash. *Genome biology* **17:** 132.

Page AJ, Cummins CA, Hunt M, Wong VK, Reuter S, Holden MT *et al* (2015). Roary: rapid large-scale prokaryote pan genome analysis. *Bioinformatics* **31:** 3691-3693.

Quast C, Pruesse E, Yilmaz P, Gerken J, Schweer T, Yarza P *et al* (2012). The SILVA ribosomal RNA gene database project: improved data processing and web-based tools. *Nucleic acids research* **41:** D590-D596.

Ritalahti KM, Amos BK, Sung Y, Wu Q, Koenigsberg SS, Löffler FE (2006). Quantitative PCR targeting 16S rRNA and reductive dehalogenase genes simultaneously monitors multiple Dehalococcoides strains. *Applied and environmental microbiology* **72:** 2765-2774.

Rodriguez-r LM, Konstantinidis KT (2013). Nonpareil: a redundancy-based approach to assess the level of coverage in metagenomic datasets. *Bioinformatics* **30:** 629-635.

Sadaiappan B, PrasannaKumar C, Nambiar VU, Subramanian M, Gauns MU (2021). Meta-analysis cum machine learning approaches address the structure and biogeochemical potential of marine copepod associated bacteriobiomes. *Scientific reports* **11:** 1-17.

Sagheddu V, Patrone V, Miragoli F, Puglisi E, Morelli L (2016). Infant early gut colonization by Lachnospiraceae: high frequency of Ruminococcus gnavus. *Frontiers in pediatrics* **4:** 57.

Sanders W, Sanders CC (1997). Enterobacter spp.: pathogens poised to flourish at the turn of the century. *Clinical microbiology reviews* **10:** 220-241.

Streit JM, Jones RN, Sader HS, Fritsche TR (2004). Assessment of pathogen occurrences and resistance profiles among infected patients in the intensive care unit: report from the SENTRY Antimicrobial Surveillance Program (North America, 2001). *International journal of antimicrobial agents* **24:** 111-118.

Suttner B, Kim M, Johnston ER, Orellana LH, Ruiz-Perez CA, Rodriguez-R LM *et al* (2021). Transcriptomic and rRNA: rDNA Signatures of Environmental versus Enteric Enterococcus faecalis Isolates under Oligotrophic Freshwater Conditions. *Microbiology spectrum* **9:** e00817-00821.

Top J, Willems R, Bonten M (2008). Emergence of CC17 Enterococcus faecium: from commensal to hospital-adapted pathogen. *FEMS Immunology & Medical Microbiology* **52:** 297-308.

WHO (2017). Global priority list of antibiotic-resistant bacteria to guide research, discovery, and development of new antibiotics.
